# B cell-derived IL-10 promotes the resolution of lipopolysaccharide-induced acute lung injury

**DOI:** 10.1038/s41419-023-05954-2

**Published:** 2023-07-13

**Authors:** Zhun Sun, Anning Chen, Hongwei Fang, Donglin Sun, Meiying Huang, Erdeng Cheng, Mengyuan Luo, Xiaoren Zhang, Hao Fang, Guojun Qian

**Affiliations:** 1 Affiliated Cancer Hospital and Institute of Guangzhou Medical University, Guangzhou, China; 2 Department of Anesthesiology, Zhongshan Hospital, Fudan University, Shanghai, China; 3 Department of Anesthesiology, Minhang Hospital, Fudan University, Shanghai, China

**Keywords:** Cytokines, Acute inflammation

## Abstract

Inflammation resolution is critical for acute lung injury (ALI) recovery. Interleukin (IL)-10 is a potent anti-inflammatory factor. However, its role in ALI resolution remains unclear. We investigated the effects of IL-10 during the ALI resolution process in a murine lipopolysaccharide (LPS)-induced ALI model. Blockade of IL-10 signaling aggravates LPS-induced lung injury, as manifested by elevated pro-inflammatory factors production and increased neutrophils recruitment to the lung. Thereafter, we used IL-10 GFP reporter mice to discern the source cell of IL-10 during ALI. We found that IL-10 is predominantly generated by B cells during the ALI recovery process. Furthermore, we used IL-10-specific loss in B-cell mice to elucidate the effect of B-cell-derived IL-10 on the ALI resolution process. IL-10-specific loss in B cells leads to increased pro-inflammatory cytokine expression, persistent leukocyte infiltration, and prolonged alveolar barrier damage. Mechanistically, B cell-derived IL-10 inhibits the activation and recruitment of macrophages and downregulates the production of chemokine KC that recruits neutrophils to the lung. Moreover, we found that IL-10 deletion in B cells leads to alterations in the cGMP–PKG signaling pathway. In addition, an exogenous supply of IL-10 promotes recovery from LPS-induced ALI, and IL-10-secreting B cells are present in sepsis-related ARDS. This study highlights that B cell-derived IL-10 is critical for the resolution of LPS-induced ALI and may serve as a potential therapeutic target.

## Background

Acute lung injury (ALI) is the pulmonary manifestation of a systemic inflammatory response syndrome that can progress to acute respiratory distress syndrome (ARDS). Although significant progress has been made in ALI pathophysiology and treatment, ALI incidence and mortality rates are still high, ranging between 30–40% [1].

Sepsis due to bacterial pneumonia is the most common risk factor for ALI [2]. When bacteria invade the lungs, they stimulate the pulmonary innate immune system and cause inflammation [3], which is a fundamental requirement to eradicate threats to the host organism. However, uncontrolled inflammation damages the lungs, weakening their barrier protection function and further triggering ARDS [4].

Neutrophils, as effector cells of ALI, are rapidly recruited to the lungs upon pathogen invasion [5]. In this process, pathogens stimulate lung macrophages or epithelial cells to produce chemokines, such as KC (keratinocyte chemoattractant, CXCL-1), CCL-2 (chemokine ligand 2), GPR-35 (G-protein-coupled receptor), and MIP-2 (macrophage inflammatory protein-2) [6, 7], which lead to the recruitment of neutrophils. Excessive lung inflammatory responses lead to neutrophil cytotoxicity, alveolar barrier damage, impairing gas exchange, and pulmonary edema [7]. Therefore, timely inflammation resolution is essential for the host to minimize lung damage and re-establish homeostasis [8].

As a critical immunosuppressive factor, interleukin (IL)-10 was initially termed as an inhibitor of cytokine synthesis [9] since it inhibits pro-inflammatory cytokines production [10]. Almost all leukocytes, including dendritic cells (DCs), monocytes/macrophages, B cells, and T cells, produce IL-10 [10]. A recent study found that IgM^+^ B cells specifically enter and populate the pulmonary microcirculation and can mitigate neutrophil inflammation via lipoxin A_4_ (LXA4) production [11]. However, the role of IL-10-secreting B cells act on the ALI resolution is still unclear.

LPS, an essential bacterial cell wall element, is frequently employed as a surrogate for infectious exposure and induces experimental murine ALI model when it is administrated into the airways [12]. We found that B cell-derived IL-10 promotes the resolution of LPS-induced ALI, primarily by inhibiting macrophage recruitment and activation, and decreasing neutrophil infiltration. Furthermore, we found that an exogenous supply of IL-10 accelerates ALI recovery. Our results highlight the essential role of B cell-derived IL-10 in the timely resolution of LPS-induced pulmonary inflammation/injury, setting up a theoretical foundation for the development of ways to treat and prevent bacteria-induced lung injury.

## Results

### IL-10 promotes recovery from LPS-induced ALI

**Fig. 1:**
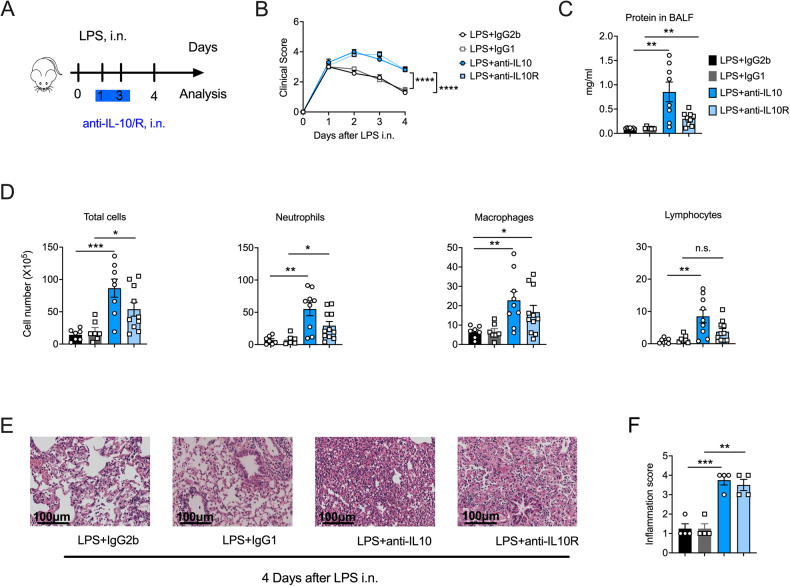
Interleukin (IL)-10 signaling blockade aggravates lipopolysaccharide (LPS)-induced acute lung injury (ALI). **A** Experimental scheme: anti-IL-10 (10 µg) or anti-IL-10 receptor (IL-10R, 50 µg) were administered intranasally on days 1 and 3 following LPS (50 μg) exposure, and IgG2b and IgG1 were as controls, respectively. The mice were euthanized on day 4 for analysis (*n* = 7–11). **B** Clinical signs of mice were observed (*n* = 7–11). **C** On day 4, post-LPS administration, BALF was collected from anti-IL-10 or anti-IL-10R (isotype control, IgG2b or IgG1) mice, and the protein concentration was determined (*n* = 7–11). **D** Differential cells in the bronchoalveolar lavage fluid were counted after Cytospin and Wright–Giemsa staining (*n* = 7–11). **E** The representative image of the HE-stained section is displayed (*n* = 4). Scale bars, 100 μm. **F** The inflammation score of the HE-stained sections. (*n* = 4). **B** Two-way ANOVA was used for the statistical analyses. **C**, **D**, **F** Student’s *t*-test was used for the statistical analyses.

IL-10 suppresses excessive immune responses to achieve homeostasis [9, 10]. To explore the role of IL-10 in ALI, we used IL-10 or IL-10 receptor (IL-10R) monoclonal antibodies to block IL-10 signaling in a murine LPS-induced ALI model (anti-IL-10/anti-IL-10R mice) and found that anti-IL-10/anti-IL-10R mice had more severe and persistent clinical symptoms (Fig. 1A, B). Moreover, compared with isotype control mice, IL-10/IL-10R blocked mice had more bronchoalveolar lavage fluid (BALF) protein, indicative of increased lung permeability (Fig. 1C), which is a critical feature of lung injury [1]. Additionally, we observed increased inflammatory cell infiltration, particularly of neutrophils (Fig. 1D), the predominant mediators of ALI [13], in IL-10/IL-10R mice. In addition, the pathological analysis demonstrated that the lungs of the anti-IL-10/anti-IL-10R ALI mice showed persistent leukocyte infiltration and severe edema, in contrast to their isotype control (Fig. 1E, F and Fig. S1A). Collectively, our results demonstrate that IL-10 facilitated the clearance of inflammatory cells and promoted alveolar repair during LPS-induced ALI.

### IL-10 inhibits prolonged lung inflammation after exposure to LPS

**Fig. 2:**
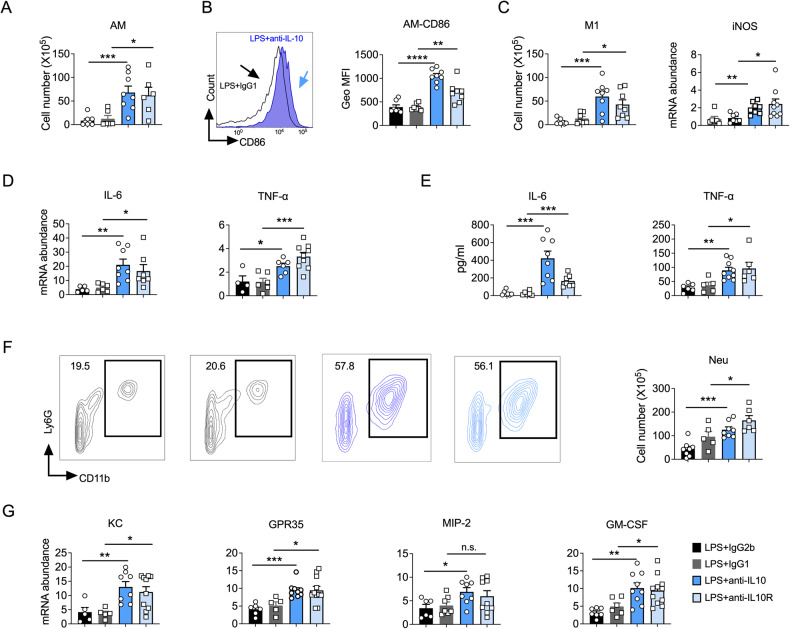
Interleukin (IL)-10 signaling blockade leads to exacerbated lung inflammation after lipopolysaccharide (LPS) exposure. **A** Flow cytometry analysis of macrophage cell numbers in mice lung (*n* = 6–8). **B** Geo MFI of CD86 on macrophages. (*n* = 6–8). **C** Flow cytometry analysis of M1 macrophage numbers in mice lung (left); RT-PCR to test the expression level of iNOS mRNA (right) (*n* = 6–10). **D** The mRNAs of IL-6 and TNF-α were detected in lung homogenates (*n* = 4–10). **E** Cytokine levels in bronchoalveolar lavage fluid samples of different groups (*n* = 5–9). **F** Neutrophils (CD11b+, Ly6G+) in the lungs as a proportion of live events. The total number of cells was then multiplied by the ratio to give the total neutrophil count. (*n* = 5–9). **G** Chemokines associated with neutrophils (*n* = 5–11). **A–G** Experiments were conducted 4 days after LPS exposure. **A–G** Student’s *t*-test was used for the statistical analyses.

Dysregulated and excessive lung inflammation is the most frequent cause of ALI [3]. Macrophages activate quickly in response to inhaled pathogens, and over-activated macrophages aggravate inflammatory responses and tissue damage [14, 15]. To further explore the impact of IL-10 act on ALI, we investigated the activation of macrophages and inflammatory cytokines involved in ALI, as previously reported [6]. On the fourth day after LPS exposure, we found that blocking IL-10 signaling enhanced the recruitment and activation of lung macrophages upon LPS stimulation (Fig. 2A, B) and increased their pro-inflammatory properties, as manifested by an elevated number of M1 macrophages and increased iNOS (nitric oxide synthase 2) expression in the lungs (Fig. 2C). However, we did not observe a difference of lung DCs, monocytes, and M2 recruitment after blocking IL-10/IL-10R during ALI (Fig. S1B, C). Moreover, the mRNA levels of IL-6 and TNF-α, which are the vital inflammatory cytokines produced by macrophages in ALI [16], were elevated after blocking IL-10/IL-10R during ALI (Fig. 2D). Similarly, the protein levels of IL-6 and TNF-α were also increased in BALF (Fig. 2E). Therefore, these results suggest that IL-10 inhibits the recruitment and activation of lung macrophages in ALI.

Neutrophil infiltration is a hallmark of lung inflammation, and neutrophil elimination is the predominant crucial sign of ALI resolution [5, 17]. Consistent with our BALF analysis (Fig. 1E), we observed an increased neutrophil infiltration in the lung (Fig. 2F) when IL-10 signaling was blocked during ALI. Neutrophil recruitment is primarily mediated by chemokines (KC [7], MIP-2 [18], GPR-35 [19], and GM-CSF [18]) produced by macrophages and epithelium [20]. Therefore, we measured the relative mRNA expression of neutrophil chemokines in the lungs of the IL-10/IL-10R mice and found that blocking IL-10 signaling increased the expression levels of these chemokines (Fig. 2G). Similar results were also obtained for BALF KC and MIP-2 levels (Fig. S1D). Collectively, these results demonstrated that IL-10 can reduce macrophage recruitment and activation in the lung and mitigate neutrophil recruitment, thereby alleviating inflammation and promoting host recovery from LPS-induced ALI.

### IL-10 is primarily produced by B cells upon LPS challenge

**Fig. 3:**
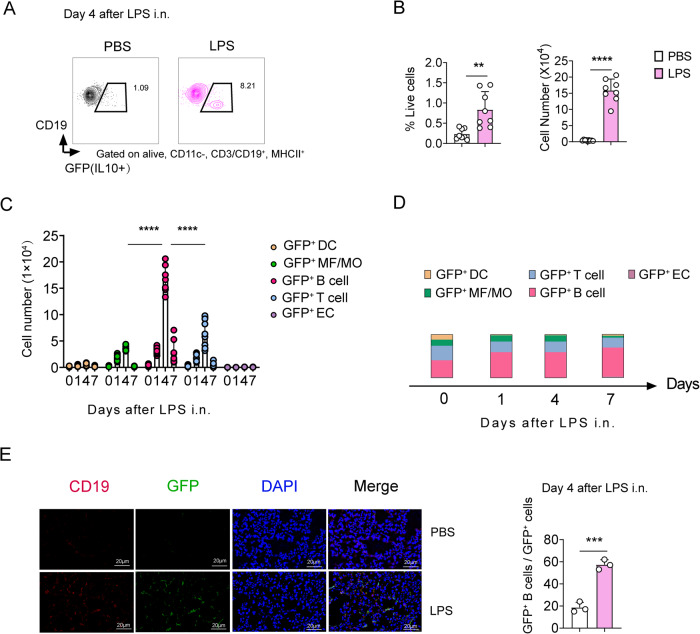
Interleukin (IL)-10 is primarily secreted by B cells during acute lung injury (ALI) resolution. **A** Flow cytometry of GFP(IL-10)^+^ B cells in the lungs. **B** Proportion and number of GFP(IL-10)^+^ B cells as a proportion of live events. The lung total number of cells was then multiplied by the ratio to give the total GFP(IL-10)^+^ B cells count. (*n* = 8). **C** The number of different GFP (IL-10)-secreting cell subsets in the lungs (*n* = 9–10); DC dendritic cells, MF macrophages, MO monocytes, EC epithelial cells. **D** The proportion of IL-10-secreting subpopulations in the lungs of IL-10-GFP mice during LPS exposure (*n* = 9–10). **E** Immunofluorescence in the lungs of mice after four days of LPS exposure and the proportion of IL-10-secreting B cells (*n* = 3). Scale bars, 20 μm. Representative one of two independent experiments. Student’s *t*-test was used for the statistical analyses.

There are numerous sources of IL-10, including macrophages, monocytes, DCs, T cells, B cells, and ECs [21]. Considering the essential role of IL-10 in ALI recovery, we used IL-10-GFP reporter mice to examine the source cell of IL-10 secretion during ALI. Thereafter, the lung IL-10^+^ (GFP^+^) cells were identified by flow cytometry using a modified gating strategy described previously (Fig. S2A, B) [22]. We found that lung IL-10-producing B cells significantly elevated after LPS stimulation and peaked at day 4, which is correlated with the resolution of ALI (Fig. 3A–C and Fig. S2C–E). Moreover, B cells produced significantly more IL-10 than other reported IL-10-producing cells, such as DCs, macrophages/monocytes (MF/Mo), T cells, and ECs after the LPS challenge (Fig. 3C, D). Immunofluorescence further confirmed these results (Fig. 3E and Fig. S2F). Altogether, these results demonstrate that IL-10 is primarily produced by B cells in LPS-induced ALI.

### B cell-derived IL-10 promotes LPS-induced ALI recovery

To investigate the role of B cells in ALI, we constructed an LPS-induced ALI model in B cell-deficient (μMT) mice. In contrast to LPS-treated WT mice, the LPS-treated μMT mice had persistent clinical signs of illness, elevated BALF protein levels, and increased inflammatory infiltrates, particularly neutrophils (Fig. S3A–D), demonstrating that B cells are essential in lung injury resolution.

**Fig. 4:**
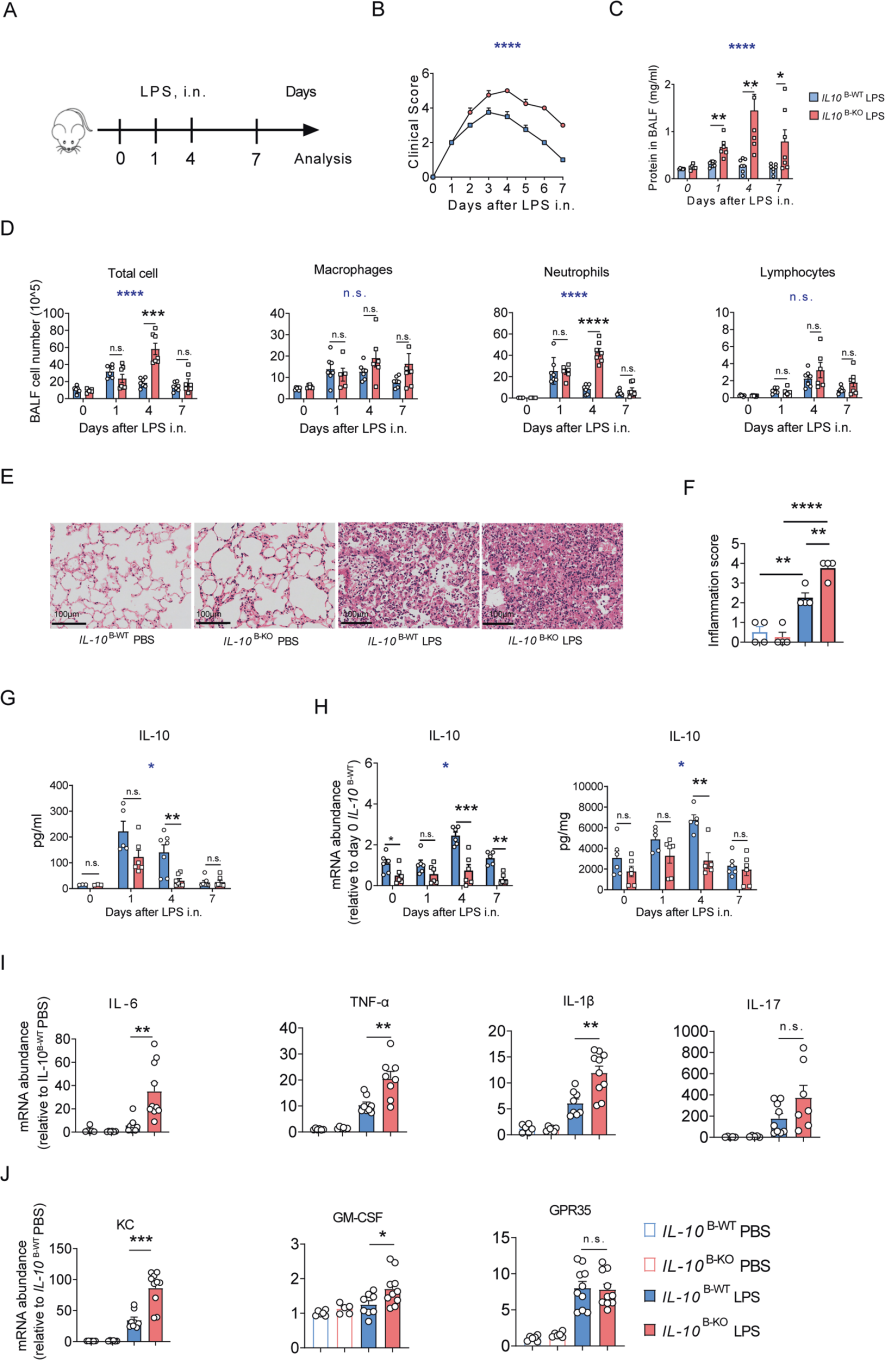
Interleukin (IL)-10 in B cells promotes acute lung injury (ALI) recovery. **A** Experimental scheme: Observation and analysis of recovery from acute lung injury in *IL-10* ^B-WT^ and *IL-1*0 ^B-KO^ mice at days 1, 4, and 7 after LPS exposure. **B** Clinical signs in mice were observed (*n* = 6–7). **C** Bronchoalveolar lavage fluid (BALF) from *IL-10* ^B-WT^ and *IL-10* ^B-KO^ mice was harvested, and the protein concentration was measured (*n* = 4–7). **D** *IL-10* ^B-WT^ and *IL-10* ^B-KO^ mice were treated with 50 μg LPS i.n., and BALF of the treated and control mice were harvested on days 1, 4, and 7. The total BALF number of cells and different types of cells were counted (*n* = 6–7). **E**, **F** The representative image of the HE-stained section and the inflammation score are displayed (*n* = 4). Scale bars, 100 μm. **G** IL-10 protein levels in BALF. **H** IL-10 mRNA and protein levels in the lung. **I** mRNAs of IL-6, TNF-α, IL1-β, and IL-17, were detected in lung homogenates (*n* = 6–8). **J** mRNAs of KC, GM-CSF, and GPR-35 were detected in lung homogenates (*n* = 6–8). **B**–**D**, **G**, **H** Two-way ANOVA was used for the statistical analyses (blue stars). **(C**, **D**, **F**–**J)** Student’s *t*-test was used for the two groups’ statistical analyses (black stars).

To further explore the role of B cell-derived IL-10 on LPS-induced ALI, we specifically deleted the IL-10 in B cells by crossing *Il10* ^fl/fl^ mice with Mb1-Cre mice (*IL10* ^*flox/flox*^*-mb1cre*, termed *IL-10* ^B-KO^), the knockout efficiency of IL-10 in B cells is demonstrated in (Fig. S4A, B) and challenged them with LPS (Fig. 4A). We found that *IL-10* ^B-KO^ mice had persistent and aggravated clinical signs, compared to the corresponding control mice (*IL-10* ^B-WT^) (Fig. 4B). Moreover, *IL-10* ^B-KO^ mice showed prolonged and increased BALF protein levels (Fig. 4C, D), indicative of long-lasting alveolar damage and decelerated tissue repair in LPS-treated *IL-10* ^B-KO^ lungs.

The total BALF cell numbers markedly increased in both *IL-10* ^B-WT^ and *IL-10* ^B-KO^ mice from day 1 post-LPS administration, primarily caused by neutrophil recruitment to the alveolar spaces (Fig. 4D). Alveolar neutrophils in *IL-10* ^B-WT^ mice were largely cleared on day 4 and eliminated on day 7, while in *IL-10* ^B-KO^ mice, alveolar neutrophils remained on day 4, suggesting that alveolar inflammation resolved more slowly in *IL-10* ^B-KO^ mice (Fig. 4D). Furthermore, the pathological analysis showed that lungs of LPS-challenged *IL-10* ^B-KO^ mice had more leukocyte infiltration and aggravated alveolar edema on day 4 (Fig. 4E, F; Fig. S4C).

Importantly, *IL-10* ^B-KO^ mice showed significantly lower BALF and lung IL-10 levels than *IL-10* ^B-WT^ mice following LPS exposure (Fig. 4G, H), further confirming that IL-10 is mainly secreted by B cells after the LPS challenge. In addition, *IL-10* ^B-KO^ mice lungs had greater mRNA levels of inflammatory cytokines (IL-6, TNF-α, and IL-1β) and neutrophil chemokines (KC, GM-CSF) compared to *IL-10* ^B-WT^ mice lungs (Fig. 4I, J). Collectively, these results indicate that B cell-derived IL-10 mitigates lung inflammation and promotes LPS-induced ALI resolution.

### IL-10 in B cells promotes ALI recovery, possibly via the cGMP–PKG pathway

**Fig. 5:**
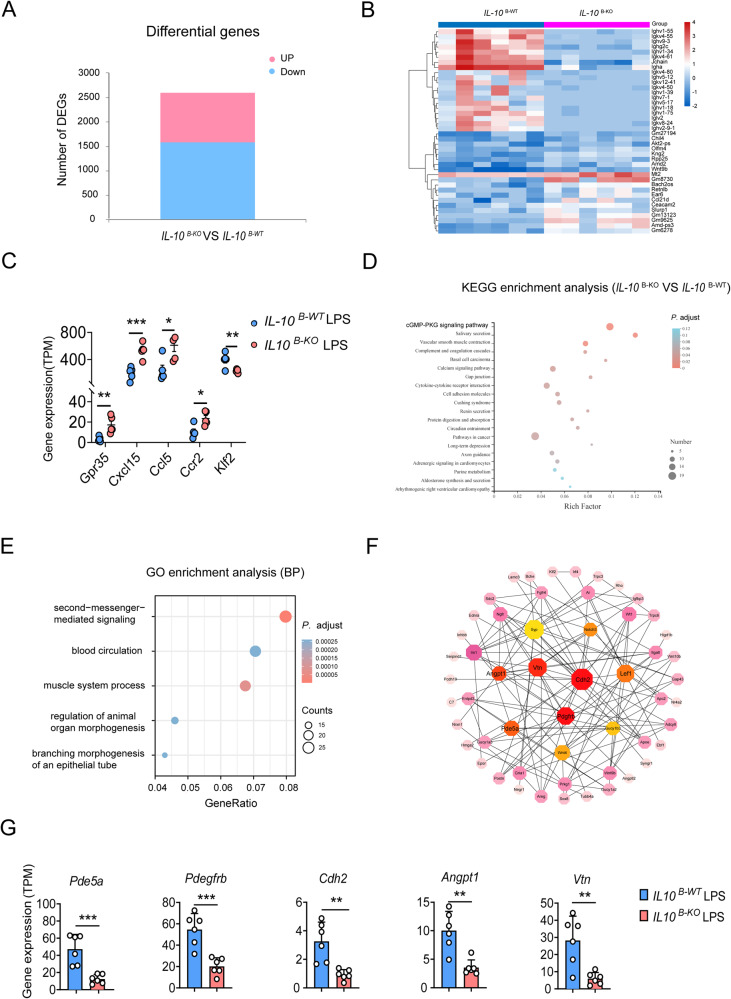
Interleukin (IL)-10 regulates PDE5A and affects the cGMP–PKG pathway in acute lung injury (ALI). **A** Gene profile changes in the lungs of *IL-10* ^B-WT^ and *IL-10* ^B-KO^ mice, analyzed by RNA-seq (*n* = 6). **B** Heatmap showing the top 20 up and down-regulated gene expression of the lung in *IL-10* ^B-WT^ and *IL-10* ^B-KO^ mice (*n* = 6). **C** Gene expression of the neutrophil-associated chemokines *Gpr35*, *Cxcl15*, *Ccl5*, *Ccr2*, and the macrophage activation-associated negative regulator *Klf2*. **D**, **E** KEGG and GO enrichment analysis. **F** Cytoscape analysis of the gene interactions network, algorithm of the network structure, and calculation of the weighted linkage between nodes to filter out the vital hub genes. **G** Expression levels of hub genes in the lung tissues of *IL-10* ^B-WT^ and *IL-10* ^B-KO^ mice (*n* = 6). Student’s *t*-test was used for the statistical analyses.

To explore the possible mechanisms involved in LPS-induced ALI recovery associated with B cell-derived IL-10, we performed RNA-Seq analysis of *IL-10* ^B-WT^ and *IL-10* ^B-KO^ mice lungs post-LPS challenge. Upon LPS challenge, *IL-10* ^B-WT^ and *IL-10* ^B-KO^ lungs had 2609 differentially expressed genes (DEGs), of which 1018 were upregulated and 1591 downregulated (Fig. 5A, B). We found that chemokines (GPR-35, CXCL15, CCL5, and CCR2) involved in the macrophages and neutrophils recruitment [19, 23–25] were significantly elevated, while the negative regulator of macrophages activation KLF2 was downregulated in *IL-10* ^B-KO^ lungs (Fig. 5C). Moreover, Kyoto Encyclopedia of Genes and Genomes (KEGG) and Gene Ontology (GO) enrichment analysis of the DEGs revealed that the cGMP–PKG pathway and second messenger-mediated signaling pathway are the most relevant signaling pathway, which plays a critical role in regulating the expression of the pro-inflammatory cytokines in macrophages [26] (Fig. 5D, E). Further analysis revealed 5 hub genes (Fig. 5F). Intriguingly, hub genes involved in angiogenesis and repair, especially *Pde5a* [27], *Pdegfrb* [28, 29] *Cdh2* [30], and *Angpt1* [31], were significantly downregulated in *IL-10* ^B-KO^ mice compared to *IL-10* ^B-WT^ mice, indicating that B cell-derived IL-10 possibly promotes resolution of lung inflammation by downregulating these critical hub genes (Fig. 5G). Notably, *Pde5a*, the primary metabolizing enzyme of cGMP, which can reduce cGMP levels [32], is downregulating in *IL-10* ^B-KO^ mice (Fig. 5G), suggesting that B-cell derived IL-10 may regulate macrophage activation through *Pde5a*–cGMP–PKG axis. These results indicate that IL-10 promotes ALI resolution, possibly via the cGMP–PKG pathway.

### An exogenous supply of IL-10 promotes the resolution of ALI

**Fig. 6:**
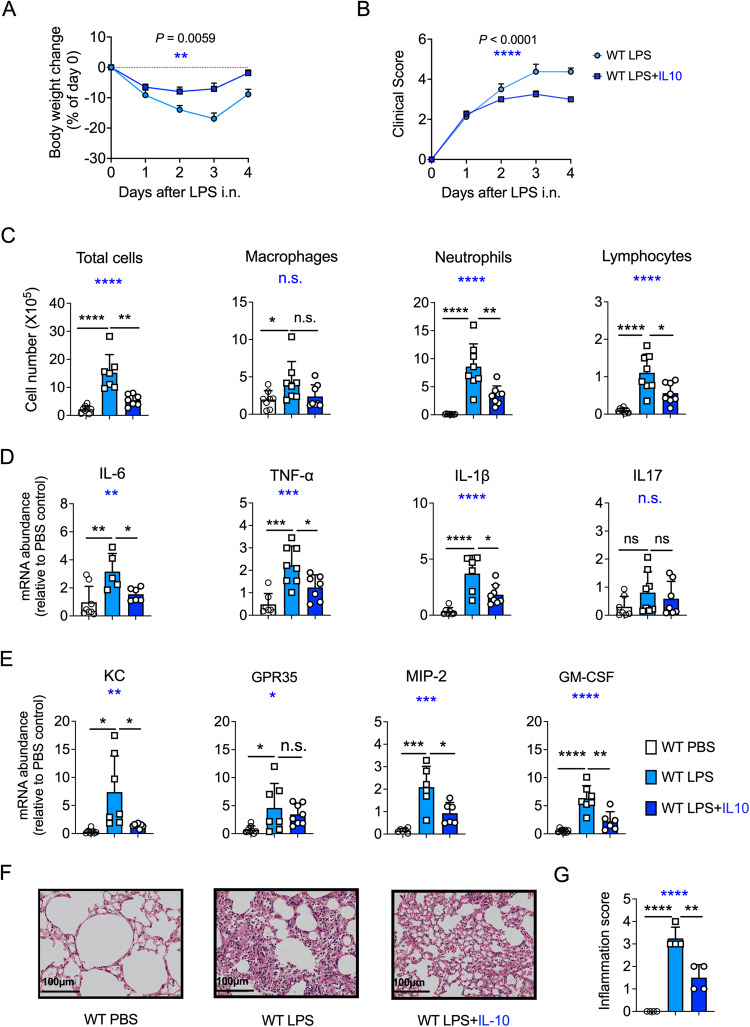
Exogenous IL-10 promotes acute lung injury (ALI) recovery. **A** Mice body weight was measured daily after intranasal (i.n.) lipopolysaccharide (LPS) administration (*n* = 8). LPS + IL-10 group was instilled with 0.1 μg of IL-10 recombinant protein on days 1 and 3 after LPS exposure. **B** Clinical signs in mice were observed (*n* = 8). **C** On day 4, after 50 μg i.n. LPS administration, bronchoalveolar lavage fluid of the WT and WT + IL-10 mice were harvested. Total cells and different types of BALF cell were counted (*n* = 8). **D** mRNA levels of IL-6, TNF-α, IL1-β, and IL-17, were detected in lung homogenates. **E** mRNA levels of KC, GPR-35, MIP-2, and GM-CSF were detected in lung homogenates. **F** The representative image of HE-stained lung sections (*n* = 4). Scale bars, 100 μm. **G** The inflammation score of the HE-stained sections (*n* = 4). **A**, **B** Two-way ANOVA was used for the statistical analyses. **C**–**E**, **G** One-way ANOVA was used for the multiple group’s statistical analyses (blue stars). Student’s *t*-test was used for the two groups’ statistical analyses (black stars).

The previous results demonstrated the critical role of IL-10 in promoting ALI resolution. Therefore, we further sought to investigate whether an exogenous supply of IL-10 displayed any therapeutic potential for ALI recovery. After supplementation with exogenous IL-10, LPS-treated mice showed rapid recovery from body weight loss, fewer clinical signs, and decreased inflammatory cell infiltration to the alveolar spaces (Fig. 6A–C). We also observed reduced cytokine and chemokine production post-exogenous IL-10 supplementation along with LPS challenge (Fig. 6D, E). Similarly, hematoxylin and eosin (HE) staining showed that supplementation with exogenous IL-10 reduced lung edema and leukocyte infiltration (Fig. 6F, G and Fig. S5A). Altogether, these results suggest that an exogenous supply of IL-10 promotes recovery from LPS-induced ALI and could be employed as a long-term ALI maintenance therapy.

### IL-10-producing B cells are present in humans with sepsis-induced ALI

**Fig. 7:**
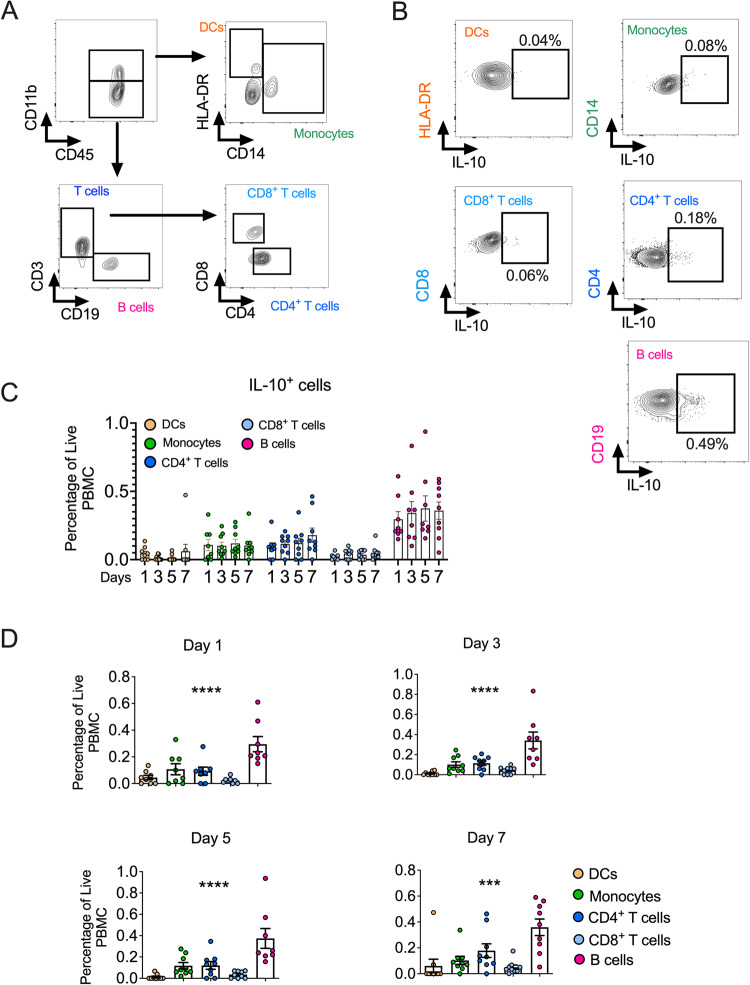
IL-10-producing B cells are present in humans with sepsis-induced ARDS. **A** Gating strategy for DCs, monocytes, B cells, and CD4^+^/CD8^+^ T cells in the peripheral blood mononuclear cells (PBMCs). **B** Flow cytometry of IL-10^+^ DCs, IL-10^+^ monocytes, IL-10^+^ CD8^+^/CD4^+^ T cells, and IL-10^+^ B cells in the PBMCs. **C**, **D** Percentage of IL-10^+^ DCs, IL-10^+^ monocytes, IL-10^+^ CD8^+^ T cells, IL-10^+^ CD4^+^ T cells, and IL-10^+^ B cells in the PBMCs over time in patients with sepsis-induced ARDS (PBMCs were collected from nine patients with sepsis-related ARDS). One-way ANOVA was used for the statistical analyses.

To study whether our findings had any effect on human ARDS, we determined whether IL-10-secreting B cells can be found in patients with ARDS. Patients with sepsis-induced ARDS were enrolled, and peripheral blood mononuclear cells (PBMCs) were harvested on days 1, 3, 5, and 7 after enrollment. We found that IL-10-secreting B cells are present in patients with sepsis-induced ARDS (Fig. 7A, B) and had a higher percentage than other PBMC cells (including DCs, monocytes, and CD4^+^/CD8^+^ T cells) (Fig. 7C, D). These findings indicate that IL-10-secreting B cells are present in the bloodstream of humans with sepsis-induced ARDS and may play a critical role in its resolution.

## Discussion

ALI is characterized by rapid alveolar-capillary injury induction, neutrophil, and macrophage infiltration-associated inflammation, and high pro-inflammatory cytokine secretion [5, 33]. The host immune system evolved to combat infection while limiting damage to the host. Although many studies investigate the role of inflammation in exacerbating ALI, little is known about how the host immune system initiates timely resolution to avoid excessive lung damage and regain lung homeostasis [34]. Furthermore, the role and the source of IL-10, a well-known anti-inflammatory cytokine, in ALI recovery are unclear. Therefore, in this study, we examined the role of IL-10 and its source cell during ALI resolution. We found that blocking IL-10 or IL-10R increased body weight loss in mice and reduced their recoverability from lung inflammation and alveolar damage, suggesting that IL-10 signaling is required for timely resolution of lung injury, consistent with the ability of IL-10 in limiting fatal tissue damage during several experimental models of infection, including *Toxoplasma gondii*, malaria, and *Trypanosoma cruzi* [21].

Macrophages and neutrophils are the most critical effector cells in lung injury [5, 35]. We found that macrophage and neutrophil infiltration to the lung and alveoli is dramatically elevated after IL-10 signaling blockade during ALI. Consistently, inflammatory cytokines secreted by macrophages (IL-6 and TNF-α) and chemokines for neutrophils recruitment (KC and GPR-35) are elevated after IL-10 signaling blockade, further exacerbating lung injury. Thus IL-10 exerts a significant anti-inflammatory effect on LPS-induced lung injury by downregulating the excessive and persistent inflammatory cell recruitment and inflammatory cytokines and chemokines secretion during ALI. This is in line with the reported ability of IL-10 in downregulating excessive inflammation in allergic airway inflammation, inflammatory bowel disease, rheumatoid arthritis, etc. [36–38].

IL-10 was discovered 30 years ago as being produced by Th2 cells [39]. Since then, several other cell types, including B cells, DCs, macrophages, and ECs, have been reported to produce IL-10 in the respiratory tract [10, 40, 41]. We tracked IL-10 production in ALI mice by using IL-10-GFP reporter mice [42], and found that IL-10 production gradually increased in the ALI process and peaked and sustained at day 4, which was correlated with the resolution of ALI. Moreover, IL-10 was primarily produced by B cells during ALI resolution. Additionally, we found that T cells are the secondary IL-10-secreting cells during ALI; however, the IL-10-secreting T cells were reported to be non-essential for ALI resolution [43]. Although IL-10 production by macrophages also has a crucial role in promoting the resolution process during LPS-induced ALI [44], we observed that macrophages is not the primary cell source of IL-10 during LPS-induced ALI. Therefore, to confirm the role of B cell-derived IL-10 in LPS-induced ALI, we constructed B cell conditional knockout IL-10 mice and found that B cell-derived IL-10 does not affect the initiation of ALI as manifested by similar infiltration of neutrophils and clinical signs between *IL-10* ^B-WT^ and *IL-10* ^B-KO^ mice following LPS stimulation at day 1. However, neutrophils were largely cleared in *IL-10* ^B-WT^ mice, along with decreased inflammatory cytokines and chemokines production at days 4 and 7, while persistent inflammation existed in *IL-10* ^B-KO^ mice. These results demonstrate the critical role of B cell-derived IL-10 in promoting inflammation resolution and hastening lung injury recovery in LPS-induced ALI.

The disruption of lung barrier structures and rupture of lung capillaries in ALI leads to altered lung permeability, resulting in increased protein accumulation in the alveoli exacerbating lung injury [45]. RNA-seq DEG analysis showed that some hub genes, such as *Pde5a* [27], *Pdgfrbβ* [29], *Cdh2* [30], *Angpt1* [31], associated with angiogenesis and wound repair healing significantly downregulated, which further suggested that B cell-derived IL-10 may influence ALI recovery by affecting angiogenesis. KEGG enrichment analysis of the DEGs revealed that they were mainly enriched in the cGMP–PKG pathway, which plays an essential role in regulating the expression of pro-inflammatory cytokines in macrophages [26]. Interestingly, *PDE5A*, a critical metabolizing enzyme of cGMP that can influence cGMP concentration in vivo [32], was down-expressed in the lungs of *IL10* ^B-KO^ mice and could affect the cGMP–PKG signaling pathway in the LPS-induced ALI model, suggesting that B-cell derived IL-10 may regulate macrophage activation through *Pde5a*–cGMP–PKG axis.

In addition, to validate that our findings have therapeutic significance in ALI treatment, we confirmed that an exogenous supply of IL-10 could alleviate inflammation and promote lung injury resolution in the murine ALI model. Thus, our results, together with other researches [46–49], demonstrating that IL-10 plays an important role in protecting the host from bacterial infection. Additionally, we demonstrated that IL-10-secreting B cells are present in humans with sepsis-induced ALI, and B cells are the predominant IL-10-producing cells in PMBCs. ARDS/ALI is a significant clinical concern, responsible for about 1.2 million deaths annually worldwide [50], and we believe that our work highlights the essential role of IL-10-secreting B cells in downregulating innate immune responses to mediate ALI resolution.

## Conclusion

Our findings suggest that B cell-derived IL-10 alleviates lung inflammation and promotes recovery from LPS-induced ALI by limiting excessive inflammatory innate immune response. In addition, our results demonstrate that IL-10-producing B cells are present in humans with ALI and may serve as a potential immunotherapeutic target.

### Limitations of the study

The methodology employed to establish B cells as the primary source of IL-10 in Fig. S2A must be considered in conjunction with the possibility of a minor constituent of monocytes, interstitial macrophages, NK cells, and neutrophils present within the defined B cell population. However, our findings were corroborated by immunofluorescence analysis and further supported by the observation of reduced IL-10 secretion in the BALF and lung following LPS exposure in mice with B cell-specific IL-10 loss. Moreover, Fig. 2F shows a potential subset of myeloid-derived suppressor cells (MDSCs) within the neutrophil gate defined by CD11b and Ly6G expression. MDSCs’ immunomodulatory function, linked to increased Arg-1 and iNOS expression [51, 52], may be involved in exacerbated lung inflammation post-anti-IL10 treatment in our model. However, additional research is required to confirm this relationship and its underlying mechanisms.

Although our study demonstrates the presence of IL-10-secreting B cells in patients with sepsis-induced ARDS and a higher percentage compared to other PBMC cells, the precise relationship between IL-10 levels in circulating PBMC and lung injury severity remains unclear. Additionally, while our RNA sequencing data suggests some signaling effects, it is important to note that this is based on whole lung sequencing and not specifically focused on macrophages. Further studies are required to fully understand the effect of IL-10 on the PKG/cGMP signaling pathway in lung macrophages.

## Materials and methods

### Study design

This research focuses on the regulatory work of IL-10 in ALI recovery, the detection of IL-10 source cells during ALI resolution, and the identification of the associated underlying immune mechanisms. For this, we inhibited IL-10 signaling in LPS-induced ALI mice by blocking IL-10/IL-10R and by using IL-10-specific loss in B-cell mice (*IL-10* ^B-KO^ mice). We observed the mortality and clinical signs; analyzed protein content in BALF; detected neutrophil, macrophage, and lymphocyte levels; conducted HE staining of the lung tissue sections; assessed lung inflammation; and analyzed lung chemokine-associated mRNA levels of these mice. Additionally, we conducted signaling pathway enrichment analysis by RNA-seq in *IL-10* ^B-KO^ mice and identified the hub genes associated with ALI resolution. Lastly, IL-10 recombinant protein was also used to evaluate the role of exogenous IL-10 in ALI resolution. All the test mice had corresponding controls. All the mice were kept under the same conditions and were age and sex-matched. In both our animal and human studies, to ensure objectivity and limit bias, blinding was implemented where feasible.

### Study population

Patient samples were selected from critically ill patients over 18 years old in the emergency intensive care units of Zhongshan Hospital, affiliated with Fudan University, and Minhang Hospital, affiliated with Fudan University, respectively. Based on the diagnostic criteria of Sepsis 3.0 proposed by the 2016 international consensus of Sepsis experts [53] and the Berlin definition and diagnostic criteria of ARDS [54], nine patients with sepsis-related ARDS were prospectively recruited. The demographic and clinical information of the study population is shown in Table 1. All procedures adhered to the Declaration of Helsinki and were authorized by the Zhongshan Hospital and Minhang Hospital Ethics Committees (ethics numbers B2022-086R and 2022-approval letter-023-01K, respectively). Each participant provided written informed permission. Our study was registered in the Clinical Trial Registry of China (registration No. ChiCTR2200060824).

**Table 1:** Clinical characteristics of patient cohorts.

Cohort:	Patients
**N**	**9**
**Demographics:**	
Age (year)	73.25 ± 6.16
Sexy (Male/Female)	2/7
**Underlying medical comorbidities,** ***N*** **(%)**	
Hypertensive disease	5(55.6)
Diabetes mellitus	3(33.3)
Coronary artery disease or congestive heart failure or myocardial infarction	4(44.4)
Chronic lung disease	6(66.7)
Cerebral infarction or Mental disorder	5(55.6)
Chronic kidney disease^**a**^	0
**Clinical information on day of enrollment:**	
Hours from hospital arrival to enrollment	20.06 ± 12.02
Hours from initial antibiotic initiation	3.39 ± 1.24
Documented T ≥ 37 °C, *N* (%)	9(100)
Documented SBP < 90 mmHg, *N* (%)	7(77.8)
Vasopressor therapy within 48 h, *N* (%)	5(55.6)
**SOFA score**^**b**^	8.22 ± 2.05
**Oxygenation index**	156.22 ± 61.24
**ARDS classification**^**c**^**, (*N*)**	
*Mild*	2
*Moderate*	5
*Severe*	2
**Clinical lab index:**	
WBC Count (*109/L)	23.37 ± 13.52
% Neutrophil	101.23 ± 40.72
% Lymphocytes	7.09 ± 5.51
% Monocytes	13.56 ± 28.07
C reactive protein (mg/l)	118.92 ± 91.78
Procalcitonin, PCT (ng/ml)	3.14 ± 2.89
Serum lactate (mmol/dL)	3.66 ± 1.96
**Endotracheal intubation,** ***N*** **(%)**	6(66.7)
**Duration of mechanical ventilation (hours)**	108.50 ± 71.86
**Clinical outcomes variables:**	
ICU stays (days)	15.67 ± 6.16
Death during index illness and/or hospitalization, *N* (%)	2(22.2)
Second hospital admission within 30 days, *N* (%)	1(14.3)
Mortality(90-day), *N* (%)	3(33.3)

All continuous values were expressed as mean ± SD.

^a^Tips: Chronic kidney disease greater than stage 3, its glomerular filtration rate <60 ml /min.

^b^Sequential Organ Failure Assessment (SOFA) score was performed on the day of admission, scoring criteria for assessing disease severity based on the functional status of six organ systems.

^c^Berlin criteria for the diagnosis of ARDS in adults.

### Blood sample collection, processing, and analysis

Peripheral blood samples (5 ml EDTA tube) were harvested at four-time points on days 1, 3, 5, and 7 after the patients were enrolled, and the blood samples were processed within 2 h to extract all PBMCs. The PBMCs with cell cryopreservation were frozen at −80 °C for subsequent flow cytometry.

Surface staining of Fc-blocked PBMCs with anti-CD45, anti-CD11b, anti-CD3, anti-CD19, anti-CD14, anti-CD4, anti-CD8, where intracellular staining was performed with anti-IL-10. Then, the FACSAria (BD Biosciences) was used to evaluate the cells, and the FlowJo program was used to analyze the resulting data (Tree Star Inc.).

### Mice

IL-10-IRES-EGFP mice have been described previously [42]. B cell-deficient μMT mice (Jackson Laboratory), *IL-10* ^*flox/flox*^ mice, and *mb1*cre mice were all bred in Zhaoqing Huaxia Kaiqi Biotechnology Co. Ltd. All of the mice had a C57BL/6 J genetic background and were raised in a specialized environment free of pathogens. *IL-10* ^B-KO^ mice, with impaired B, cell-derived IL-10 secretion, were obtained by mating *IL-10* ^*flox/flox*^ with *mb1-*cre mice. The Animal Ethics Committee of Guangzhou Medical University approved all animal experiments (Project number: 2019-273; S2023-154; Casgene-202212010057). Approximately 150 mice were used for the relevant study. Each protocol was in accordance with the Guide for the Care and Use of Laboratory Animals.

### Reagents

We bought LPS (*Escherichia coli* O111:B4; L2630-100MG) from Sigma. Types of anti-mouse antibodies used in flow cytometry were as follows: anti-CD45 (30-F11), anti-CD206 (MR6F3), anti-CD11c (N418), anti-CD11b (M1/70), anti-MHC-II (M5/114.15.2), anti-CD86 (GC1), anti-CD19 (1D3), anti-CD3 (145-2C11), anti-DAPI, and anti-CD326 (clone G8.8). RNAi Plus (Cat# 9190) and RNA inversion kit was purchased from Takara; Enzyme-Linked Immunosorbent Assay (ELISA) kit for IL-6, TNF-α, KC, and MIP-2 ELISA kits were obtained from BD and R&D. Purified anti-IL-10 (JES5-16E3) and anti-IL-10R (1B1.3a) were purchased from Invitrogen and BioLegend, respectively. Recombinant murine IL-10 (cat# 200-10-2) was obtained from PeproTech.

### Lung injury models

WT, *IL-10* ^B-WT^, *IL-10* ^B-KO^, μMT mice, and GFP(IL-10) *Tiger* mice were instilled with LPS (50 μg) or PBS. Mice were sacrificed on days 0, 1, 4, and 7. BALF was obtained after lung perfusion with PBS, and the lungs were subsequently excised for further analysis.

In experiments aimed at blocking IL-10, mice were anesthetized using pentobarbital. On the first- and third-days following exposure to LPS, intranasal administration of 10 µg of anti-IL-10 or mouse IgG2b isotype (eB149/10H5, eBioscience) was performed. In parallel, experiments aimed at blocking the IL-10 receptor (IL-10R) involved the intranasal administration of 50 µg of anti-IL-10R or IgG1 isotype (Ebrg1, Invitrogen) on days 1 and 3 post-LPS exposure.

For experiments utilizing recombinant interleukin-10 (IL-10), mice were initially anesthetized with pentobarbital and then randomly divided into three groups. Two of these groups were subjected to intranasal administration of LPS to induce ALI, while the third group served as a control and was administered PBS. Among the ALI model groups, one was treated with 0.1 µg of IL-10 recombinant protein on days 1 and 3.

### Clinical symptom score

Mice were weighed daily before and after receiving 50 μg of *E. coli* LPS, and their morbidity was assessed quantitatively by a blinded method. Each symptom received a one-point clinical score, which comprised weight loss (>10%), rash, oozing eyes, shortness of breath, and fatigue.

### BALF analysis

Mice were sacrificed, and their BALF was harvested twice by gentle perfuse of the lungs with 1 mL PBS. A bicinchoninic acid (BCA) kit to determine the protein level of the BALF supernatant was used (Pierce). Additionally, different cell types were counted using Cellometer (Nexelcom) after Cytospin and Wright–Giemsa staining. The percentage distribution of neutrophils, lymphocytes, and macrophages was assessed by counting 300 cells in each sample and calculating the total number of each cell type in BALF.

### Flow cytometry and sorting

Mouse lungs were excised, cut into pieces, and placed into culture tubes containing 5 ml digestion buffer with 1 mg/ml IV collagenase and 5 U/ml DNaseI. Lung tissue was digested and filtered via a 70 mm cell filter. Next, the cells were extracted, and the total number of cells was counted.

Anti-mouse CD16/32 was incubated with collected cells for 40 min at 4 °C to prevent nonspecific binding. Following the staining of the cells with antibodies, cell type analysis was performed using FACSAria (BD Biosciences). To sort B cells in lung mice, we incubated single cell suspension with anti-CD45, anti-CD19, and anti-MHCII, then sorted on BD FASAriaIII.

### Real time-PCR

Utilizing RNAiso Plus (TaKaRa) to separate the total RNA from the lung tissues, reverse transcription was carried out using an RNA inversion kit (TaKaRa). As an internal control, actin was used, and the relative expression of genes was evaluated using the ΔΔCt quantification and expressed as fold-changes in contrast to PBS-treated controls.

### RNA-Seq analysis

Gene expression levels were computed using transcripts per million reads (TPM). Gene richness was measured using RNA-Seq by Expectation-Maximization. DEGs analysis was performed using the DESeq2 [55]/DEGseq [56]/edgeR [57]/Limma/NOIseq [58], DEGs with a fold of change ≥1 and *P* adjust value ≤0.05 (DESeq2 /edgeR /Limma) /*P* adjust ≤0.001 (DEGseq) /Prob >0.8 (NOIseq) considered to be significant DEGs. GO and KEGG functional analyses were also carried out to determine which DEGs were significantly enriched in GO and KEGG pathways at *P* adjust <0.05 compared to the full transcriptome.

### Histological analysis

Mice lung fixed with 4% paraformaldehyde was sectioned and stained with HE. The samples were then analyzed under a Nikon E200 microscope for inflammatory cell infiltration, alveolar edema, and tissue destruction. Inflammation score measures inflammatory cell infiltration in the primary bronchus and surrounding major blood vessels, rated as follows: 0: No cells; 1: Minimal cells; 2: Unevenly distributed cells; 3: Abundant, evenly distributed cells; 4: Significant clustering of cells. The total lung injury score is the sum of these ratings, verified by experienced pathologists.

### Immunofluorescence analysis

IL-10 GFP mice were subjected to LPS infection, and after 4 days, lung tissue was harvested for subsequent preparation as cryosections. The sections were then stained with anti-CD19 antibody and analyzed via immunofluorescence microscopy utilizing a Zeiss microscope.

### Statistical analysis

An unpaired two-tailed Student’s *t*-test was used to examine group differences using PRISM (GraphPad, San Diego, CA, USA). One-way ANOVA was used for the comparison among multiple groups of statistical analyses. The two-way ANOVA test was used to compare two trends over time. Statistical significance was set at *P* < 0.05, and data were presented as means ± standard error of the mean (SEM). *P < 0.05; **P < 0.01; ***P < 0.001; ****P < 0.0001; n.s. not significant.

## Supplementary information

checklist

Supplementary file

Original Data File

Figure S1

Figure S2

Figure S3

Figure S4

Figure S5

## Data Availability

All the information required to determine and assess the conclusions in the study is provided in the paper or supplemental resources. Raw sequencing data have been deposited in the Gene Expression Omnibus (GEO) repository with the accession code GSE236391.
