# Supplementary material for: B cell-derived IL-10 promotes the resolution of lipopolysaccharide-induced acute lung injury
Source: Cell Death Dis. 2023 Jul 13;14(7):418. doi: 10.1038/s41419-023-05954-2 (PMC10345008; doi:10.1038/s41419-023-05954-2)
Supplement: Supplementary file 2 — Supplementary file [file 41419_2023_5954_MOESM2_ESM.docx]

**Supplementary Figures Legends**

**Fig. S1. Interleukin (IL)-10 signaling blockade leads to exacerbated lung inflammation.**

**(A)** The remaining images depicting HE stained sections of mouse lung tissue for Figure 1E. Scale bars, 100 μm. **(B)** Gating strategy for macrophage (MF), M1 macrophage (M1), M2 macrophage (M2), dendritic cell (DC), monocytes (Mono), and neutrophils (Neu). **(C)** DC, Mono, and M2 numbers in the lungs (n = 6–8). **(D)** BALF level of Neutrophil chemokines in anti-IL-10 or anti-IL-10R mice (n = 6–8). Student's t-test was used for the statistical analyses.

**Fig. S2. LPS-induced lung injury in GFP(IL-10) *Tiger* mice.**

**(A, B)** Gating strategy for dendritic cell (DC), macrophage (MF)/monocyte (MO), T cell, B cell, and epithelial cell (EC) populations in the lung. **(C, D)** We continuously measured Mice's Body weight changes and clinical signs for one week after LPS exposure (n = 6). **(E)** The total BALF number of cells were counted on the 0, 1, 4, and 7 days after LPS exposure (n = 5–6). **(F)** The remaining images illustrating the immunofluorescence analysis of mouse lung tissue after four days of LPS exposure for Figure 3E. Scale bars, 20 μm.

**Fig. S3. Delayed recovery of acute lung injury (ALI) in B-cell-deficient mice.**

**(A)** The clinical symptoms of mice were observed (n = 9). **(B)** On days 1, 4, and 7 after 50 μg i.n. LPS administration and bronchoalveolar lavage fluid (BALF) of the WT and μMT mice were harvested, and the protein level was determined (n = 8–9). **(C, D)** Total cell numbers and different cell types were measured in BALF (n = 8–9). **(A, B, C, D)** Two-way ANOVA was used for the statistical analyses (blue stars). **(B, C, D)** Student's t-test was used for the two groups statistical analyses (black stars).

**Fig. S4. Deleted the IL-10 in B cells by crossing *Il10* ^fl/fl^ mice with Mb1-Cre mice.**

**(A).** Mice lung cells were harvested and suspended for incubation with DAPI and a suite of antibodies including anti-CD45, anti-CD19, and anti-MHCII. Subsequently, a flow cytometry-based FACS sorting method was employed on a FACSAria III platform to isolate B cells for downstream analysis. **(B).** Isolated B cells underwent analysis for the relative mRNA expression of IL-10 using RT-qPCR (n = 6). **(C).** The remaining images depicting HE stained sections of mouse lung tissue for Figure 4E. Scale bars, 100 μm. Student's t-test was used for the two groups statistical analyses.

**Fig. S5. Exogenous IL-10 administration alleviates lung injury in mice.**

**(A).** The remaining images depicting HE stained sections of mouse lung tissue for Figure 6F. Scale bars, 100 μm.
