# Supplementary material for: B cell-derived IL-10 promotes the resolution of lipopolysaccharide-induced acute lung injury
Source: Cell Death Dis. 2023 Jul 13;14(7):418. doi: 10.1038/s41419-023-05954-2 (PMC10345008; doi:10.1038/s41419-023-05954-2)
Supplement: Supplementary file 3 — Original Data File [file 41419_2023_5954_MOESM3_ESM.docx]

This study does not involve Western blots.
