# Supplementary figures and images for: B cell-derived IL-10 promotes the resolution of lipopolysaccharide-induced acute lung injury
Source: Cell Death Dis. 2023 Jul 13;14(7):418. doi: 10.1038/s41419-023-05954-2 (PMC10345008; doi:10.1038/s41419-023-05954-2)

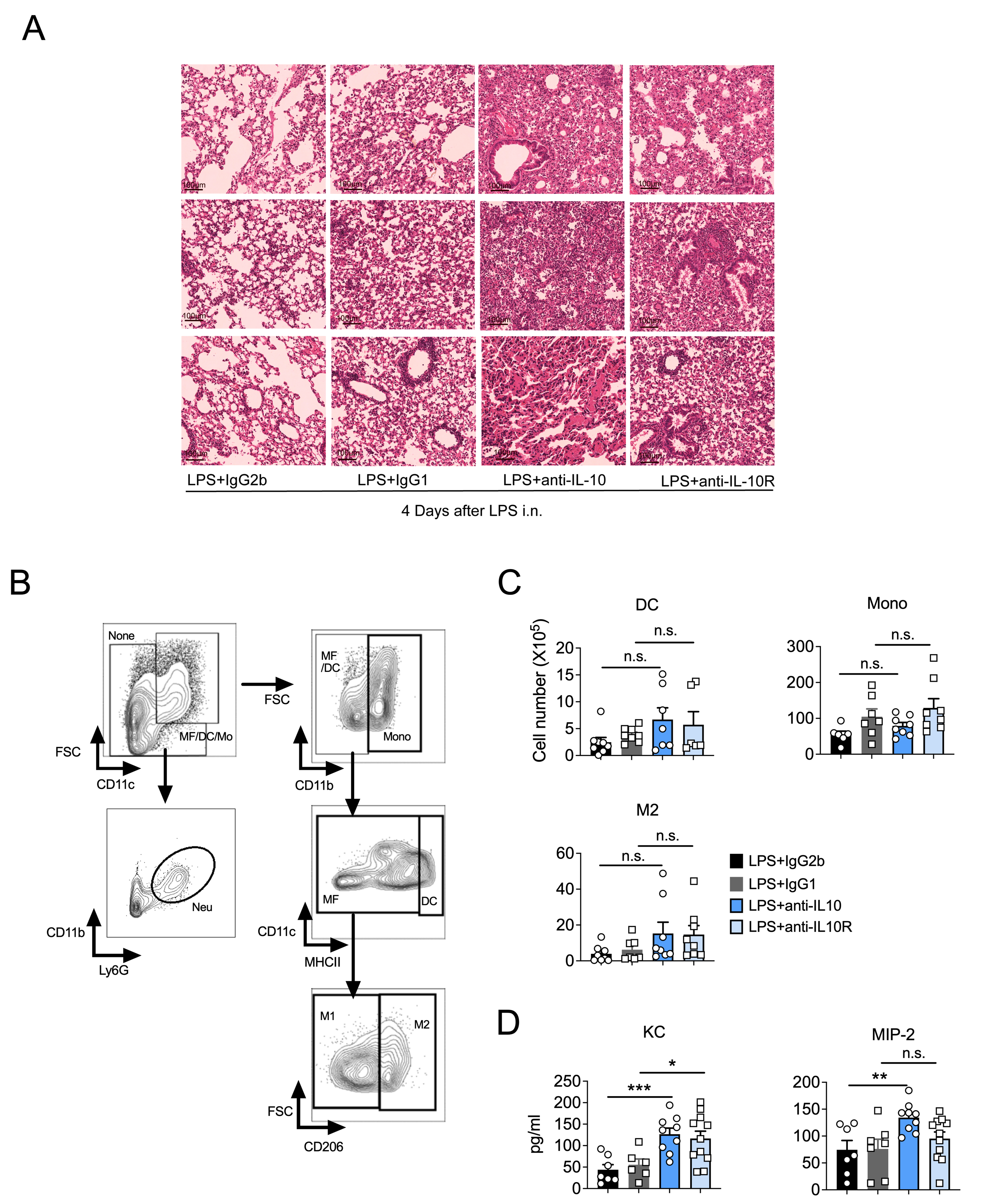

Supplement: Supplementary file 4 — Figure S1 [file 41419_2023_5954_MOESM4_ESM.tif]

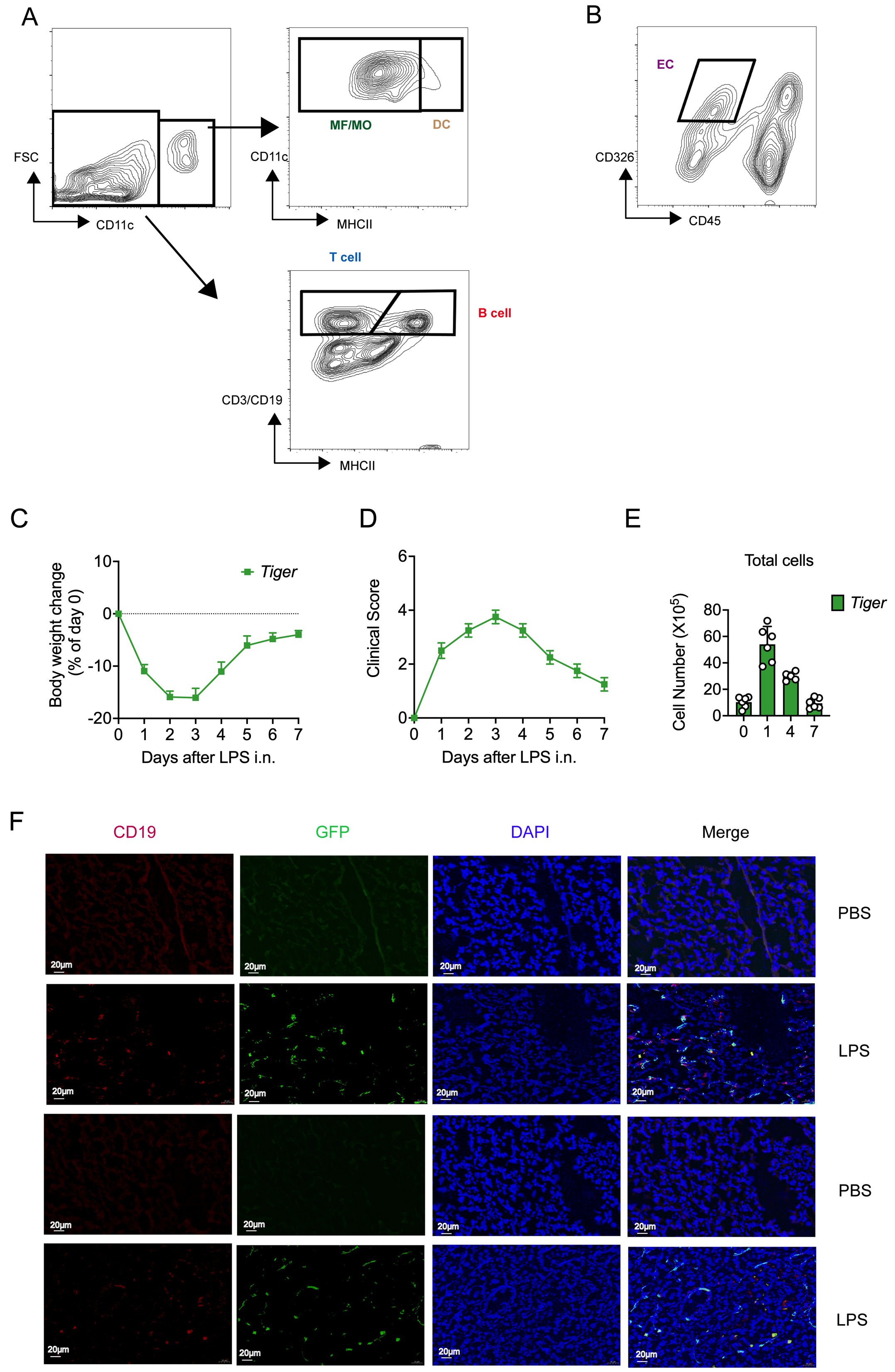

Supplement: Supplementary file 5 — Figure S2 [file 41419_2023_5954_MOESM5_ESM.tif]

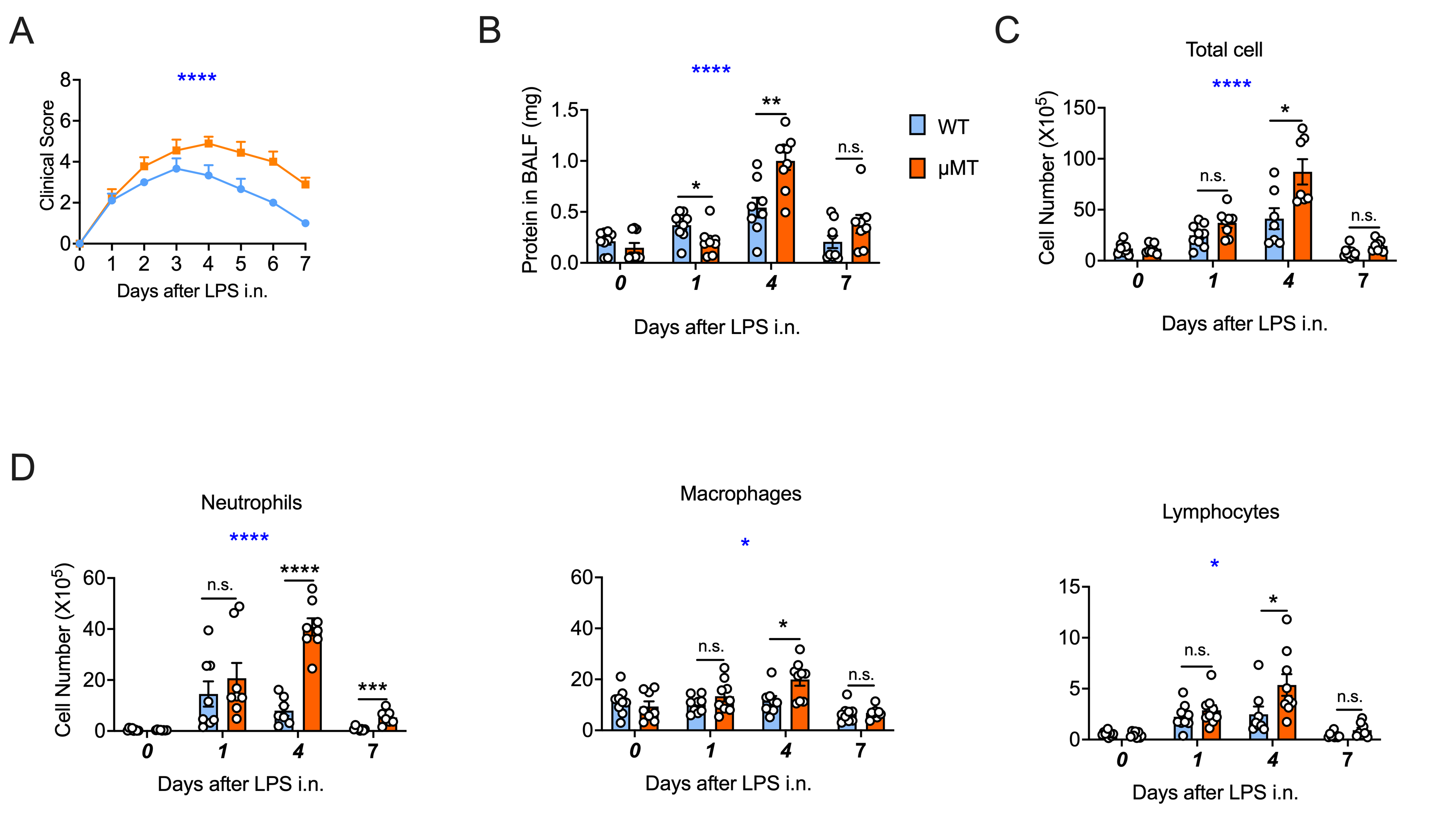

Supplement: Supplementary file 6 — Figure S3 [file 41419_2023_5954_MOESM6_ESM.tif]

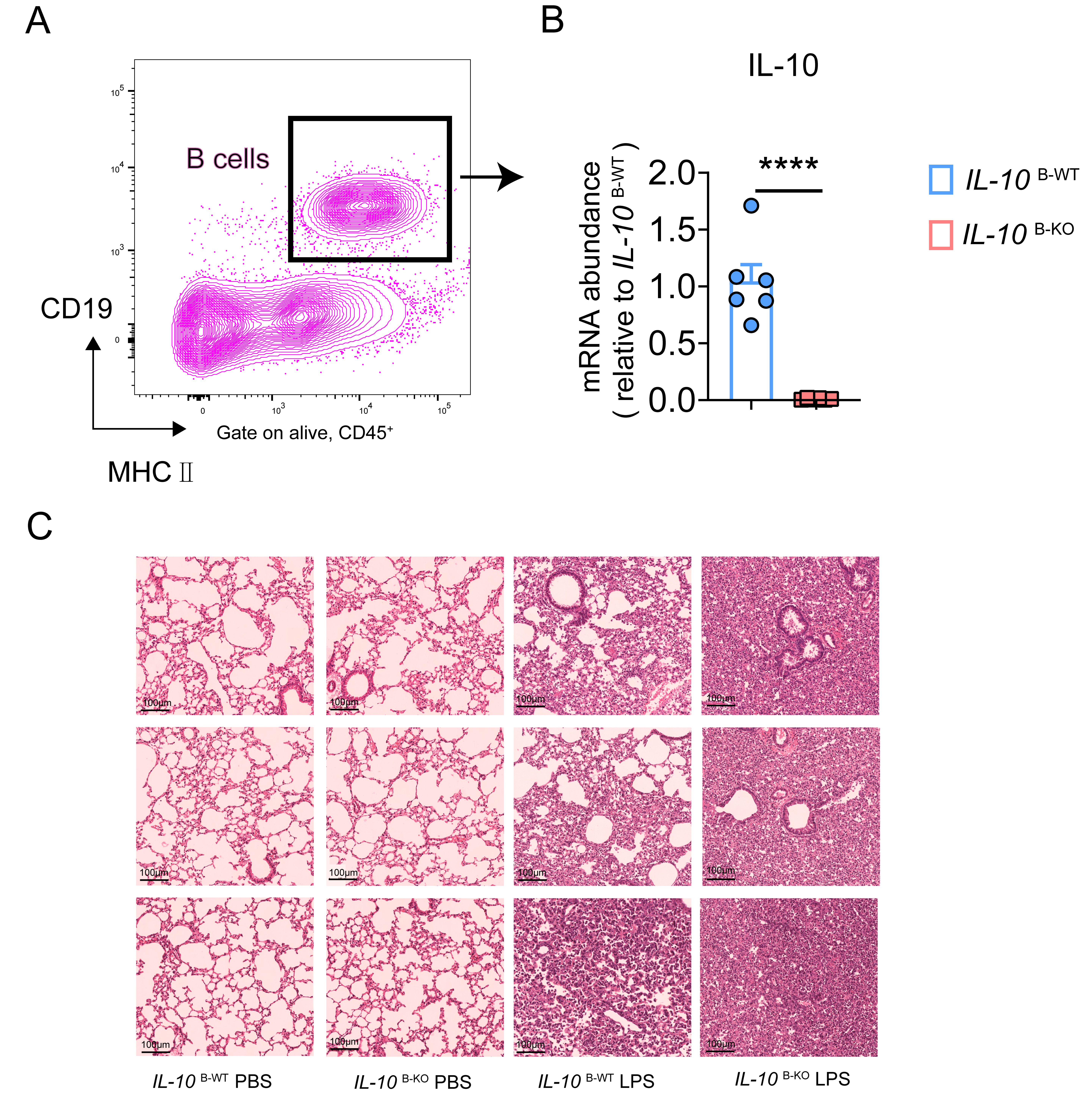

Supplement: Supplementary file 7 — Figure S4 [file 41419_2023_5954_MOESM7_ESM.tif]

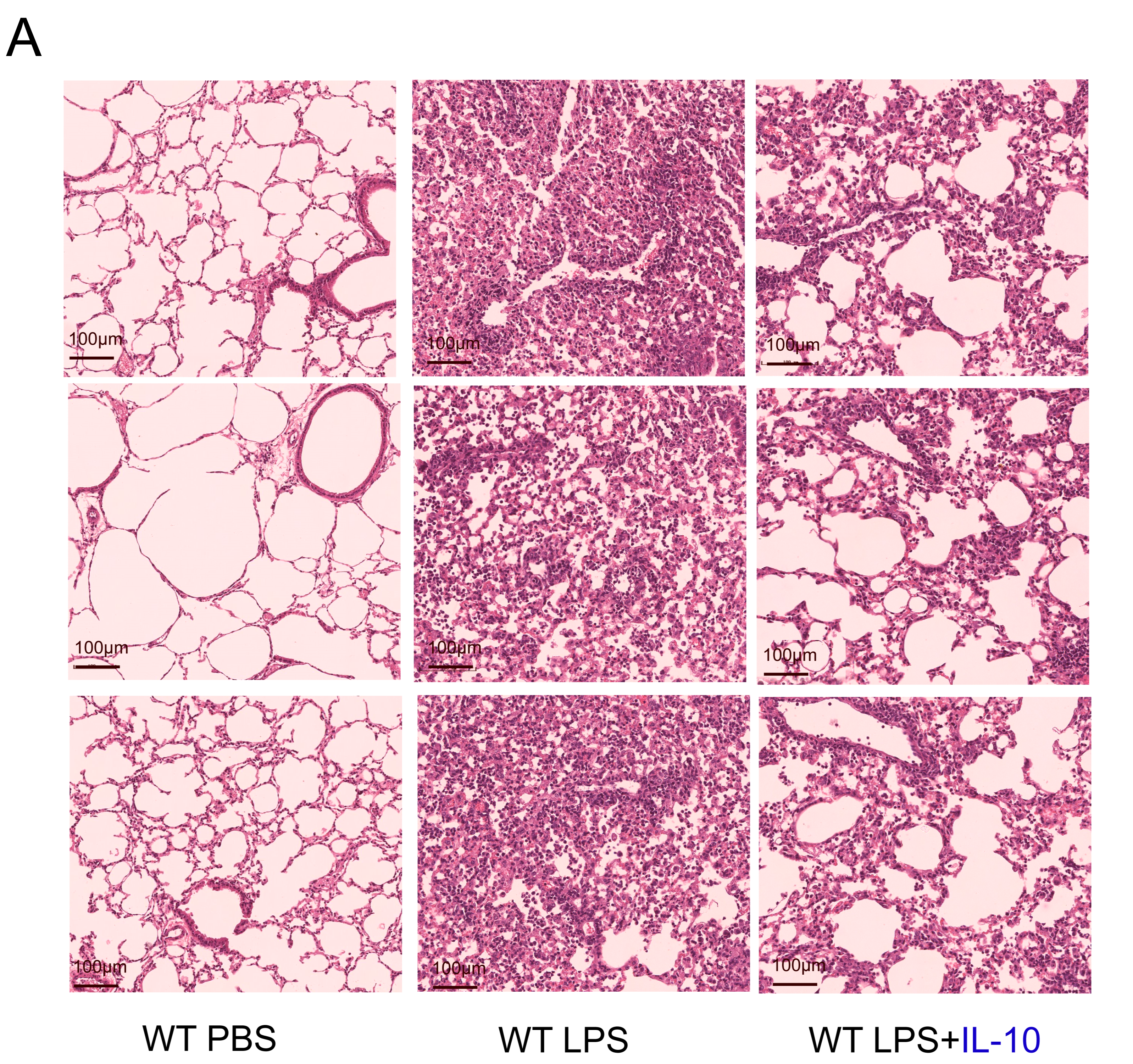

Supplement: Supplementary file 8 — Figure S5 [file 41419_2023_5954_MOESM8_ESM.tif]
